# Diagnostic value and clinical significance of Ki-67, PI3K, and Fascin in patients with oral squamous cell carcinoma

**DOI:** 10.5937/jomb0-54944

**Published:** 2025-11-05

**Authors:** Yang Guanglian

**Affiliations:** 1 The Second Affiliated Hospital of Guizhou Medical University, Kaili City, Qiandongnan Miao and Dong Autonomous Prefecture, Guizhou Province, 556000, China

**Keywords:** oral squamous cell carcinoma, Ki-67, phosphoinositide-3-kinase, Fascin, biomarkers, prognosis, tumour metastasis, immunohistochemistry, oralni skvamozni karcinom, Ki-67, fosfoinozitid-3-kinaza, Fascin, biomarkeri, prognoza, metastaze tumora, imunohistohemija

## Abstract

**Background:**

Oral squamous cell carcinoma (OSCC) is a common and aggressive malignancy that poses significant challenges in terms of diagnosis and prognosis. Identifying reliable biomarkers for early detection and prognostication is essential for improving patient outcomes. Ki-67, PI3K, and Fascin are potential biomarkers involved in OSCC progression, but their combined diagnostic value and clinical significance remain underexplored. Objective: This study aims to evaluate the expression levels of Ki-67, PI3K, and Fascin in OSCC tissues and investigate their associations with clinical and pathological parameters, including tumour differentiation, cervical lymph node metastasis, and TNM staging.

**Methods:**

A retrospective analysis was conducted on 35 patients diagnosed with OSCC, with tumour tissues subjected to immunohistochemical staining to assess the expression of Ki-67, PI3K, and Fascin. The association between marker expression and clinical features was evaluated, and correlations between the markers were analysed.

**Results:**

The expression levels of Ki-67, PI3K, and Fascin were significantly higher in OSCC tissues compared to paracancerous tissues (P&lt; 0.05). Higher expression levels of these markers were associated with well-differentiated tumours and cervical lymph node metastasis (P&lt; 0.05). However, no significant differences were found between early and advanced tumour stages (P&gt; 0.05). Strong positive correlations were observed between Ki-67, PI3K, and Fascin expression, indicating potential shared molecular pathways involved in OSCC progression.

**Conclusions:**

Ki-67, PI3K, and Fascin are promising biomarkers for diagnosing and predicting the prognosis of OSCC. Their combined assessment could aid in early detection, identification of high-risk patients, and determination of appropriate treatment strategies. Further research is needed to fully understand the molecular mechanisms underlying their role in OSCC progression and to assess their clinical utility in patient management.

## Abstract (sr)

**Uvod:**

Oralni skvamozni karcinom (OSCC) je česta i agresivna maligna bolest koja predstavlja značajne izazove u dijagnostici i prognozi. Identifikacija pouzdanih biomarkera za ranu detekciju i prognoziranje bolesti je ključna za poboljšanje ishoda kod pacijenata. Ki-67, PI3K i Fascin su potencijalni biomarkeri uključeni u progresiju OSCC-a, ali njihova zajednička dijagnostička vrednost i klinički značaj ostaju nedovoljno istraženi. Cilj: Cilj ove studije je da se proceni nivo ekspresije Ki-67, PI3K i Fascina u tkivima OSCC-a i ispita njihova povezanost sa kliničkim i patološkim parametrima, uključujuci diferencijaciju tumora, metastaze u cervikalne limfne čvorove i TNM stadijum.

**Metode:**

Sprovedena je retrospektivna analiza kod 35 pacijenata dijagnostikovanih sa OSCC-om, pri čemu su tumorska tkiva podvrgnuta imunohistohemijskom bojenju radi procene ekspresije Ki-67, PI3K i Fascina. Analizirana je povezanost između ekspresije markera i kliničkih karakteristika, kao i korelacije između samih markera.

**Rezultati:**

Nivoi ekspresije Ki-67, PI3K i Fascina su bili značajno viši u OSCC tkivima u poređenju sa tkivima u okolini tumora (P&lt; 0,05). Viši nivoi ekspresije ovih markera su bili povezani sa dobro diferenciranim tumorima i pri - sustvom metastaza u cervikalnim limfnim čvorovima (P&lt; 0,05). Međutim, nisu nađene značajne razlike između ranih i uznapredovalih stadijuma tumora (P &gt; 0 ,0 5 ). Uočene su jake pozitivne korelacije između ekspresije Ki67, PI3K i Fascina, što ukazuje na moguce zajedničke molekularne puteve u progresiji OSCC-a.&nbsp;

**Zaključak:**

Ki-67, PI3K i Fascin predstavljaju obecavajuce biomarkere za dijagnozu i prognozu OSCC-a. Njihova zajednička procena može pomoci u ranoj detekciji, identifikaciji pacijenata sa visokim rizikom i određivanju adekvatnih terapijskih strategija. Potrebna su dalja istraživanja kako bi se u potpunosti razumeli molekularni mehanizmi njihove uloge u progresiji OSCC-a i procenila njihova klinička primena u lečenju pacijenata.

## Introduction

Oral squamous cell carcinoma (OSCC) is a prevalent and aggressive malignancy that arises from the epithelial lining of the oral cavity [1]. It is responsible for significant morbidity and mortality worldwide, with its incidence rates steadily increasing in various regions, especially among individuals who engage in risky behaviours such as tobacco use, alcohol consumption, and poor oral hygiene [2]. Despite advances in diagnostic and therapeutic modalities, the prognosis of OSCC patients remains suboptimal, primarily due to delayed diagnosis, frequent lymphatic metastasis, and the ability of tumours to invade surrounding tissues. Consequently, the identification of novel biomarkers for early detection, prognostication, and therapeutic intervention is crucial for improving patient outcomes [3] [4].

Ki-67, a nuclear protein associated with cell proliferation, is widely recognised as a biomarker of tumour cell growth and is commonly used to assess the proliferation index of various cancers, including OSCC. Its expression correlates with the aggressiveness of the tumour and may provide valuable insights into the tumour's potential for invasion and metastasis. Similarly, phosphoinositide-3-kinase (PI3K), a key regulator of cellular processes such as growth, survival, and metabolism, plays a pivotal role in the pathogenesis of numerous cancers, including OSCC. The PI3K pathway has been implicated in promoting cancer cell proliferation, survival, and resistance to treatment. The aberrant activation of this signalling pathway is associated with advanced tumour stages and poor prognosis, making it a promising target for therapeutic strategies [5] [6].

Fascin, an actin-bundling protein, is involved in cellular migration, invasion, and the formation of cellular protrusions, all of which are essential for cancer metastasis. It is overexpressed in a variety of cancers, including OSCC, and has been linked to tumour progression and poor prognosis [7]. Fascin's role in promoting invasive behaviours in cancer cells, particularly in the context of oral cancer, makes it an attractive candidate for evaluation as a diagnostic and prognostic biomarker.

The diagnostic and clinical significance of Ki-67, PI3K, and Fascin in OSCC remains an area of active research. While previous studies have demonstrated their individual roles in tumour progression, few have evaluated their combined diagnostic utility or their correlation with key clinical outcomes such as tumour differentiation, lymph node metastasis, and TNM staging [8] [9] [10]. This study aims to investigate the expression levels of Ki-67, PI3K, and Fascin in OSCC tissues and their potential correlations with clinical and pathological parameters, such as tumour differentiation, cervical lymph node metastasis, and TNM staging. Furthermore, the study explores the prognostic significance of these markers in predicting tumour invasiveness and patient outcomes [10] [11] [12] [13].

In this context, we hypothesise that the combined assessment of Ki-67, PI3K, and Fascin expression can provide a more comprehensive and reliable diagnostic approach, aiding in the identification of high-risk patients who may benefit from more aggressive treatment strategies. Additionally, the study seeks to provide further insight into the molecular mechanisms underlying OSCC pathogenesis, which could lead to the development of targeted therapies aimed at inhibiting these critical biomarkers to improve patient survival and quality of life. Through the integration of these biomarkers into clinical practice, we aim to enhance early detection, better understand the disease's biological behaviour, and ultimately improve the management of OSCC.

## Materials and methods

### Research participants

The clinical data of patients diagnosed with oral squamous cell carcinoma (OSCC) who were consecutively admitted to our department between January 2022 and March 2024 were retrospectively reviewed. All patients included in the study had not received any form of radiotherapy, chemotherapy, or biological treatment prior to surgery and had complete clinical records. A total of 35 cases were included, comprising 22 male and 28 female patients, aged between 27 and 78 years, with a median age of 54.8 years. Tumour locations included 15 cases in the buccal mucosa, 2 cases in the gingiva, 3 cases in the floor of the mouth, and 15 cases in the tongue. According to the 1997 edition of the World Health Organisation's histological classification of oral cancer, 16 cases were classified as well-differentiated, 13 as moderately differentiated, and 6 as poorly differentiated. Based on the tumour-node-metastasis (TNM) staging system, 5 cases were staged as I, 12 as stage II, 11 as stage III, and 7 as stage IV Of the total, 17 patients had cervical lymph node metastasis, while 18 did not.

### Inclusion criteria

Pathologically confirmed diagnosis of OSCC.

No prior radiotherapy or chemotherapy before surgery.

Complete clinical data available.

Patients with other primary cancers were excluded from the study. Additionally, paracancerous tissue samples, obtained from areas more than 2 cm away from the cancerous tissue, were collected as controls, with negative margins confirmed by pathology.

### Specimen preparation and immunohistochemical staining

The pathological specimens of the 35 OSCC cases were paraffin-embedded tissue samples obtained from the Department of Pathology at our hospital. Sections of 4 μm thickness were prepared using a microtome. Immunohistochemical staining was performed using the EnVision method, following the kit instructions, and all sections were processed under identical conditions. The detailed procedure is as follows:

Routine deparaffinisation of sections with water and washing 3 times with PBS for 5 minutes each.

Seal with 3% H_2_O_2 _in distilled water or PBS at room temperature for 10 minutes, followed by 3 PBS washes of 3-5 minutes each.

Antigen retrieval, as per antibody requirements, followed by 3 PBS washes of 3-5 minutes each.

Block with normal goat serum (or horse serum, 5% BSA, etc.) for 20 minutes at room temperature, discarding excess liquid.

Incubate sections in the primary antibody for 12 hours at 37°C or overnight at 4°C (after overnight incubation, sections should be reheated at 37°C for 1 hour the next day), followed by 3 PBS washes of 5 minutes each.

Add the secondary antibody dropwise and incubate for 20-30 minutes at 20-37°C, followed by 3 PBS washes of 3-5 minutes each.

Colour development using a DAB kit or selfmade colour reagent was monitored under a microscope and stopped with PBS.

Counterstaining with hematoxylin or methyl green for the nucleus, followed by differentiation in a solution, washing with tap water, and bluing.

Dehydrate, clear with clearing agents, and mount the sections.

Ki-67 staining was assessed by positive nuclear staining. Scoring was as follows: 1+: less than 10% of cells positive; 2 + : 10-50% positive; 3 + : more than 50% positive. PI3K and Fascin were evaluated in the cytoplasm and/or nucleus of epithelial cells, with positive staining indicated by brown-yellow particles. Staining was scored based on the proportion of positive cells: S 25% (1 point), 26%-50% (2 points), and > 50% (3 points). Staining intensity was scored as 0 (unstained), 1 (light yellow), 2 (yellow), or 3 (brown). The final score was calculated by multiplying the proportion of stained cells by the intensity score, with scores < 4 considered negative and ≥ 4 positive.

### Detection of Ki-67, PI3K, and Fascin

The levels of Ki-67, PI3K, and Fascin were detected using enzyme-linked immunosorbent assay (ELISA). A total of 0.1 mL of appropriately diluted anti-Ki-67, PI3K, or Fascin antibodies were added to the wells of a polystyrene reaction plate, capped, and incubated at 4°C for 24 hours. The plate was then washed three times and spin-dried. Subsequently, 0.1 mL of diluted test specimens (prepared in 0.02 mol/L Tris-HCl buffer, pH 7.4), as well as positive and negative control specimens, were added to each well and incubated at 43°C for 60 minutes. After removing the liquid, the plate was washed three times and spin-dried again. A substrate solution (5.14 mL of 0.1 mol/L Na_2_HPO_4_ and 4.86 mL of 0.05 mol/L citric acid) was added to each well, followed by 0.1 mL of o-phenylenediamine and incubation in the dark for 20 minutes. The reaction was stopped with 0.05 mL of 2 mol/L H_2_SO_4_, and the absorption value at A405 was measured using a microplate reader to assess the levels of Ki-67, PI3K, and Fascin.

### Statistical processing

Data analysis was performed using SPSS version 23.0. Categorical data were presented as percentages and intergroup comparisons were conducted using the χ^2^ test. For multi-group comparisons, the F test was applied. Pearson's correlation coefficient was used to analyse the relationships between variables. A p-value of < 0.05 was considered statistically significant.

## Results

### Expression of Ki-67, PI3K, and Fascin in OSCC and paracancerous tissues

As presented in Table 1, the expression levels of Ki-67, PI3K, and Fascin were significantly higher in OSCC (oral squamous cell carcinoma) tissues compared to paracancerous tissues, with the difference being statistically significant (P<0.05). This suggests that these markers are more prominently expressed in cancerous tissues, potentially indicating their role in OSCC progression.

**Table 1:** Comparison of expression rates of Ki-67, PI3K, and Fascin in OSCC tissues and paracancerous tissues (n (%)).

Grouping	n	Ki-67	PI3K	Fascin
Cancer<br>tissues	35	32 (91.42)	30 (85.71)	29 (82.85)
Paracancerous<br>tissues	35	4 (11.42)	5 (14.28)	3 (8.57)
χ^2^		44.837	35.714	38.914
P		0.000	0.000	0.000

### Association between Ki-67, PI3K, and Fascin expression and OSCC differentiation

Table 2 demonstrates that the positive expression rates of Ki-67, PI3K, and Fascin were notably higher in well-differentiated OSCC tissues than in moderately or poorly differentiated OSCC tissues. This finding indicates a correlation between the expression of these proteins and the level of tumour differentiation. In well-differentiated tumours, which tend to grow more slowly and retain some normal cell functions, the higher expression of these markers may reflect increased cellular proliferation and signalling activity, contrasting with the reduced activity in more poorly differentiated tumours, which are more aggressive and disorganised.

**Table 2:** Correlation of Ki-67, PI3K and Fascin expression with differentiation of OSCC.

Grouping	n	Ki-67	PI3K	Fascin
Well-differentiated	16	15 (93.75)	16 (100)	15 (93.75)
Moderately<br>differentiated	13	7 (53.84)	6 (46.15)	6 (46.15)
Poorly-differentiated	6	1 (16.67)	2 (33.33)	1 (16.67)
χ^2^		12.801	13.822	13.577
P		0.002	0.001	0.001

### Association between Ki-67, PI3K, and Fascin expression and cervical lymph node metastasis in OSCC patients

According to Table 3, OSCC patients with cervical lymph node metastasis exhibited higher positive expression rates of Ki-67, PI3K, and Fascin compared to those without metastasis (P<0.05). This suggests that the overexpression of these proteins may be associated with the metastatic potential of OSCC. Since lymph node metastasis is a common and critical factor in the prognosis of OSCC, the elevated levels of these markers in metastatic cases could indicate their role in promoting tumour spread and aggressiveness.

**Table 3:** Correlation of Ki-67, PI3K and Fascin expression with cervical lymph node metastasis in patients with OSCC.

Grouping	n	Ki-67	PI3K	Fascin
Cervical lymph<br>node metastasis	17	16 (94.11)	15 (88.23)	17 (100)
No cervical<br>lymph node<br>metastasis	18	2 (11.11)	1 (5.55)	3 (16.66)
χ^2^		24.115	24.083	24.792
P		0.000	0.000	0.000

### Association between Ki-67, PI3K, and Fascin expression and TNM staging in OSCC patients

As shown in Table 4, no statistically significant difference was found in the positive expression rates of Ki-67, PI3K, and Fascin between OSCC patients in stages I and II versus those in stages III and IV (P>0.05). This result suggests that the expression of these markers may not be closely tied to the tumour stage in terms of TNM classification, which is a system used to describe the extent of cancer spread. While these proteins are elevated in OSCC, their levels may not vary significantly as the tumour progresses from early to more advanced stages, potentially indicating that other factors, apart from the stage, may influence their expression.

**Table 4:** Correlation of Ki-67, PI3K and Fascin expression with TNM staging in patients with OSCC.

Grouping	n	Ki-67	PI3K	Fascin
Stage I + II	17	14 (82.35)	12 (70.58)	15 (88.23)
Stage III + IV	18	13 (72.22)	11 (61.11)	14 (77.77)
c2		0.509	0.349	0.673
p		0.476	0.555	0.412

### Association between Ki-67, PI3K, and Fascin expression and perifocal muscle invasion in OSCC patients

As shown in Table 5, OSCC patients who had perifocal muscle invasion displayed significantly higher positive expression rates of Ki-67, PI3K, and Fascin compared to those without muscle invasion (P<0.05). This suggests that the overexpression of these markers may be linked to the invasive behaviour of OSCC, particularly in its ability to invade adjacent muscle tissues. The higher expression of these proteins in tumours with perifocal muscle invasion could indicate a more aggressive cancer phenotype that facilitates tissue invasion.

**Table 5:** Correlation of Ki-67, PI3K and Fascin expression with perifocal muscle invasion in patients with OSCC.

Perifocal<br>muscle	n	Ki-67	PI3K	Fascin
Yes	17	13 (76.47)	15(88.23)	14 (82.35)
No	18	4 (22.22)	3(16.67)	5 (27.78)
χ^2^		10.300	17.927	10.493
P		0.001	0.000	0.001

### Correlation analysis of Ki-67, PI3K, and Fascin expression

**Figure 1:**
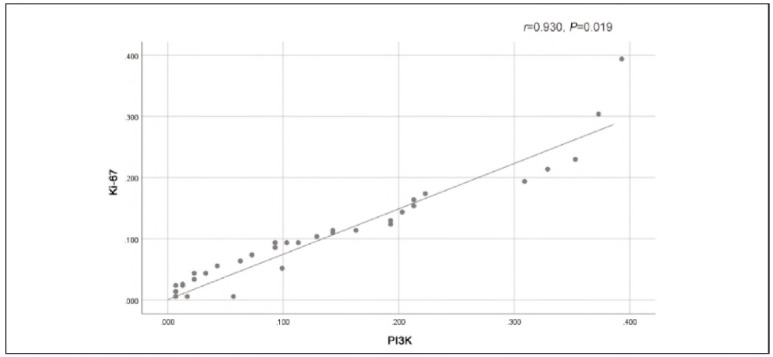
Positive correlation between Ki-67 and PI3K.

**Figure 2:**
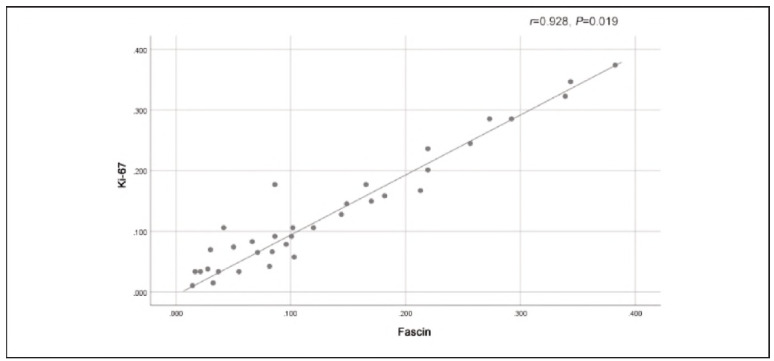
Positive correlation between Ki-67 and Fascin.

**Figure 3:**
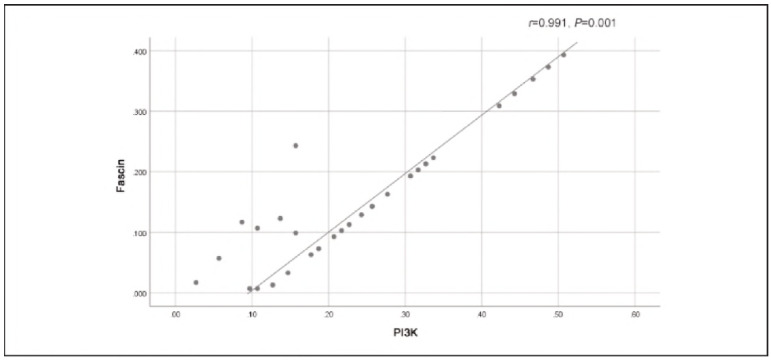
Positive correlation between PI3K and Fascin.

Figure 1, Figure 2 and Figure 3 illustrate that there were strong positive correlations between Ki-67 and PI3K (r=0.930, P=0.019), Ki-67 and Fascin (r=0.928, P=0.019), and Fascin and PI3K (r=0.991, P=0.001). These findings indicate that the expression of these markers is closely related to each other, suggesting that they might work together in the molecular pathways involved in OSCC progression. The high correlation between these proteins, particularly between Fascin and PI3K, supports the idea that they might share standard regulatory mechanisms or play complementary roles in promoting tumourigenesis and metastasis in OSCC.

In summary, these results high-light the potential of Ki-67, PI3K, and Fascin as biomarkers for OSCC diagnosis and prognosis, as their expression correlates with factors such as differentiation, metastasis, muscle invasion, and potentially the molecular dynamics driving cancer progression.

## Discussion

The present study evaluated the diagnostic value and clinical significance of Ki-67, PI3K, and Fascin in patients with oral squamous cell carcinoma (OSCC), and its findings provide important insights into their potential roles as biomarkers in OSCC progression. Our results demonstrate that the expression levels of Ki-67, PI3K, and Fascin are significantly elevated in OSCC tissues compared to paracancerous tissues, indicating their involvement in the tumourigenesis of OSCC. Furthermore, we observed that higher expression levels of these proteins were associated with more differentiated tumours and cervical lymph node metastasis. Still, no significant differences were noted between early (stage I-II) and advanced (stage III-IV) tumour stages. This suggests that Ki-67, PI3K, and Fascin might be linked to the proliferative and metastatic potential of OSCC, making them valuable candidates for prognostic biomarkers. Additionally, strong positive correlations between these markers further support the hypothesis that they may act through shared molecular pathways involved in OSCC progression.

In line with previous research, our findings highlight the critical role of Ki-67 as a proliferation marker, with several studies confirming its association with poor prognosis in various cancers, including OSCC [14] [15]. Ki-67 has been widely utilised to evaluate tumour aggressiveness, as its expression correlates with higher tumour grade and metastatic potential, as seen in our results where well-differentiated tumours exhibited higher Ki-67 levels than poorly differentiated ones. Similarly, the role of PI3K in cancer progression, particularly in head and neck cancers, has been well-documented [14] [16]. Our study aligns with these studies, as PI3K expression was significantly elevated in OSCC tissues with cervical lymph node metastasis, suggesting its involvement in the metastatic spread of OSCC. Fascin, an actin-bundling protein, has also emerged as an important player in cancer cell invasion and metastasis. Several studies have established its association with poor prognosis in multiple cancers, including OSCC [17] [18]. Our findings complement these studies, as higher Fascin expression correlated with invasive behaviour, such as perifocal muscle invasion.

Interestingly, while previous research has suggested that the expression of these markers may vary significantly across different tumour stages [19], our study did not find a significant correlation between Ki-67, PI3K, or Fascin expression and TNM staging. This could suggest that, although these markers are important for predicting tumour aggression and metastasis, they may not be closely related to the extent of cancer spread as defined by the TNM system. This observation warrants further investigation into the molecular mechanisms underlying OSCC progression, which may not solely be dictated by tumour size or stage.

Our study has several limitations that must be considered when interpreting the results. First, the sample size was relatively small, with only 35 cases included, which may limit the generalizability of our findings. Second, the retrospective design of the study does not allow for the assessment of these biomarkers' predictive power over time, especially in terms of long-term patient survival and recurrence. Third, although we found significant associations between Ki-67, PI3K, and Fascin expression and clinical outcomes such as differentiation and metastasis, the molecular pathways through which these markers influence OSCC progression remain to be fully elucidated. Future studies with larger sample sizes, prospective designs, and more comprehensive molecular analyses are needed to confirm the utility of these biomarkers in OSCC diagnosis and prognosis.

In conclusion, the combined assessment of Ki-67, PI3K, and Fascin expression provides valuable diagnostic and prognostic information in patients with OSCC. These markers, particularly when used together, have the potential to enhance early detection, predict tumour aggressiveness, and inform treatment strategies. However, further research is necessary to understand the molecular roles of these proteins fully and to determine their clinical application in OSCC management.

## Dodatak

### Conflict of interest statement

All the authors declare that they have no conflict of interest in this work.

## References

[1] Jung K, Kang H, Mehra R (2018). Targeting phosphoinositide 3-kinase (PI3K) in head and neck squamous cell carcinoma (HNSCC). Cancers Head Neck.

[2] Lakshminarayana S, Augustine D, Rao R, Patil S, Awan K, Venkatesiah S, Haragannavar V, Nambiar S, Prasad K (2018). Molecular pathways of oral cancer that predict prognosis and survival: A systematic review. J Carcinog.

[3] Cai Y, Dodhia S, Su G H (2017). Dysregulations in the PI3K pathway and targeted therapies for head and neck squamous cell carcinoma. Oncotarget.

[4] Szturz P, Vermorken J B (2020). Management of recurrent and metastatic oral cavity cancer: Raising the bar a step higher. Oral Oncol.

[5] Lamb M C, Tootle T L (2020). Fascin in Cell Migration: More Than an Actin Bundling Protein. Biology (Basel).

[6] Liu H, Zhang Y, Li L, Cao J, Guo Y, Wu Y, Gao W (2021). Fascin actin-bundling protein 1 in human cancer: Promising biomarker or therapeutic target?. Mol Ther Oncolytics.

[7] Pfisterer K, Jayo A, Parsons M (2017). Control of nuclear organization by F-actin binding proteins. Nucleus.

[8] Almangush A, Heikkinen I, Mäkitie A A, Coletta R D, Läärä E, Leivo I, Salo T (2017). Prognostic biomarkers for oral tongue squamous cell carcinoma: A systematic review and meta-analysis. Br J Cancer.

[9] Kiyohara T, Miyamoto M, Shijimaya T, Nagano N, Nakamaru S, Makimura K, Tanimura H (2018). Pseudovascular squamous cell carcinoma: A review of the published work and reassessment of prognosis. J Dermatol.

[10] Liu L, Xue X, Xue L (2019). Liver metastatic basaloid squamous cell carcinoma with negative expression of pancytokeratin: A case report and literature review. Diagn Pathol.

[11] Qiu X, Meng Y, Lu M, Tian C, Wang M, Zhang J (2021). Primary squamous cell carcinoma of the pancreas with a large pseudocyst of the pancreas as the first manifestation: A rare case report and literature review. BMC Gastroenterol.

[12] Roslan N H, Makpol S, Mohd Y Y A (2019). A Review on Dietary Intervention in Obesity Associated Colon Cancer. Asian Pac J Cancer Prev.

[13] Pirozzi F, Ren K, Murabito A, Ghigo A (2019). PI3K Signaling in Chronic Obstructive Pulmonary Disease: Mechanisms, Targets, and Therapy. Curr Med Chem.

[14] Ijaz S, Akhtar N, Khan M S, Hameed A, Irfan M, Arshad M A, Ali S, Asrar M (2018). Plant derived anticancer agents: A green approach towards skin cancers. Biomed Pharmacother.

[15] Zhou Y, Hu W, Chen P, Abe M, Shi L, Tan S, Li Y, Zong L (2017). Ki67 is a biological marker of malignant risk of gastrointestinal stromal tumors: A systematic review and meta-analysis. Medicine (Baltimore).

[16] Kiupel M, Camus M (2019). Diagnosis and Prognosis of Canine Cutaneous Mast Cell Tumors. Vet Clin North Am Small Anim Pract.

[17] Elmaci Ä, Altinoz M A, Bolukbasi F H, Yapicier O, Sav A (2017). Paradoxical results obtained with Ki67-labeling and PHH3-mitosis index in glial tumors: A literature analysis. Clin Neuropathol.

[18] Miettinen M M, Antonescu C R, Fletcher C D M, Kim A, Lazar A J, Quezado M M, Reilly K M, Stemmer-Rachamimov A, Stewart D R, Viskochil D, Widemann B, Perry A (2017). Histopathologic evaluation of atypical neurofibromatous tumors and their transformation into malignant peripheral nerve sheath tumor in patients with neurofibromatosis 1: A consensus overview. Hum Pathol.

[19] Rindi G, Klimstra D S, Abedi-Ardekani B, Asa S L, Bosman F T, Brambilla E, Busam K J, de Krijger R R, Dietel M, El-Naggar A K, Fernandez-Cuesta L, Klöppel G, McCluggage W (2018). A common classification framework for neuroendocrine neoplasms: An International Agency for Research on Cancer (IARC) and World Health Organization (WHO) expert consensus proposal. Mod Pathol.

